# Supplementary material for: Promotion of Melanoma Cell Proliferation by Cyclic Straining through Regulatory Morphogenesis
Source: Int J Mol Sci. 2022 Oct 6;23(19):11884. doi: 10.3390/ijms231911884 (PMC9569601; doi:10.3390/ijms231911884)
Supplement: Supplementary file 1 [file ijms-23-11884-s001.zip › Supplementray Informations.pdf]

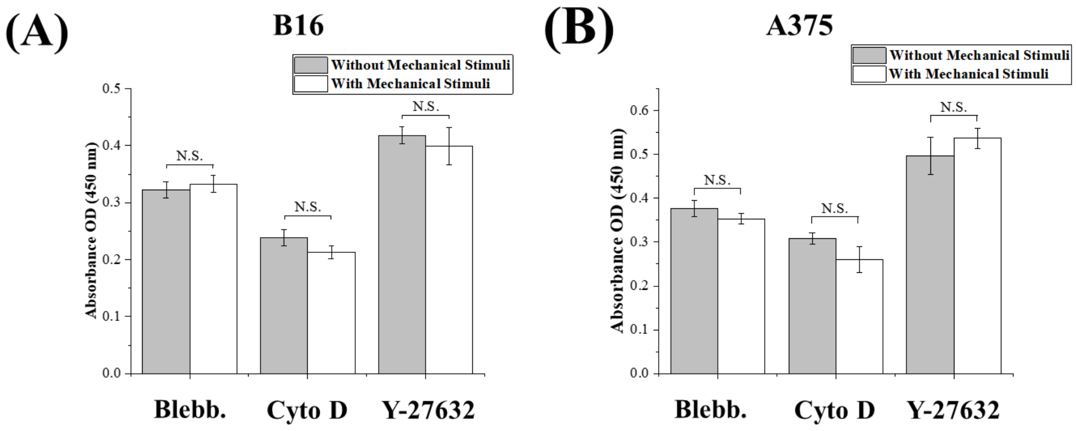

Figure S1 Influence of cyclic straining and inhibitors on melanoma cells' proliferation. (A) and (B) Quantitative data of melanoma cells' proliferation from CCK-8 assay. Data are presented as means ± SDs (n = 3).
